# Carbapenem Non-Susceptible Enterobacteriaceae in Quebec, Canada: Results of a Laboratory Surveillance Program (2010–2012)

**DOI:** 10.1371/journal.pone.0125076

**Published:** 2015-04-24

**Authors:** Brigitte Lefebvre, Simon Lévesque, Anne-Marie Bourgault, Michael R. Mulvey, Laura Mataseje, David Boyd, Florence Doualla-Bell, Cécile Tremblay

**Affiliations:** 1 Laboratoire de santé publique du Québec, Institut national de santé publique du Québec, Sainte-Anne-de-Bellevue, Québec, Canada; 2 McGill University Health Centre and Department of Medicine, McGill University, Montréal, Québec, Canada; 3 Bacteriology and Enteric Diseases Program, National Microbiology Laboratory, Public Health Agency of Canada, Winnipeg, Manitoba, Canada; 4 Centre de Recherche du Centre Hospitalier de l'Université de Montréal, Montréal, Québec, Canada; Universidad de Granada, SPAIN

## Abstract

The emergence and spread of carbapenemase-producing Enterobacteriaceae (CPE) represent a major public health concern because these bacteria are usually extensively resistant to most antibiotics. In order to evaluate their dissemination in Quebec, a surveillance program was introduced in 2010. We report the molecular and epidemiological profiles of CPE isolates collected. Between August 2010 and December 2012, a total of 742 non-duplicate isolates non-susceptible to carbapenems were analysed. AmpC β-lactamase and metallo-β-lactamase production were detected by Etest and carbapenemase production by the modified Hodge test (MHT). Antibiotic susceptibility profiles were determined using broth microdilution or Etest. Clonality of *Klebsiella pneumoniae* carbapenemase (KPC) strains was analyzed by pulsed-field gel electrophoresis (PFGE). The presence of genes encoding carbapenemases as well as other β-lactamases was detected using PCR. Of the 742 isolates tested, 169 (22.8%) were CPE. Of these 169 isolates, 151 (89.3%) harboured a *bla*_KPC_ gene while the remaining isolates carried *bla*_SME_ (n = 9), *bla*_OXA-48_ (n = 5), *bla*_NDM_ (n = 3), and *bla*_NMC_ (n = 1) genes. Among the 93 KPC strains presenting with a unique pattern (unique PFGE pattern and/or unique antibiotics susceptibility profile), 99% were resistant to ertapenem, 95% to imipenem, 87% to meropenem, 97% to aztreonam, 31% to colistin and 2% to tigecycline. In 19 patients, 2 to 5 KPC strains from different species or with a different PFGE pattern were isolated. CPE strains were present in the province of Quebec with the majority of strains harbouring KPC. Alternately, SME, OXA-48 and NMC containing strains were rarely found.

## Introduction

Carbapenems (i.e. ertapenem, meropenem, imipenem, doripenem) are bactericidal antibiotics of the β-lactam family. The emergence of carbapenemase-producing Enterobacteriaceae (CPE) is a major health concern because these bacteria are resistant to multiple classes of antibiotics that can lead to therapeutic failure [1]. Carbapenem resistance has been associated with different mechanisms including carbapenemase production, overexpression of chromosomal AmpC and porin mutations or ESBL production combined to porin mutations. Enterobacteriaceae harbouring carbapenemases belong to one of three classes according to the Ambler classification system [2]: class A serine β-lactamases (i.e. *Klebsiella pneumoniae* carbapenemase [KPC], *Serratia marcescens* enzyme [SME], not metalloenzyme carbapenemase [NMC]), class B metallo-β-lactamase (i.e. New Delhi metallo-beta-lactamase [NDM]) and the class D oxacillinase (i.e. oxacillin-hydrolyzing [OXA-48]). Carbapenemase genes are known to have chromosomal or plasmid localization [2]. Bacterial transmission of carbapenemase genes is related to the transfer of mobile genetic elements such as plasmids or transposons [2] thereby facilitating outbreaks.

The first strain of *K*. *pneumoniae* producing KPC carbapenemase was identified in North Carolina, United States of America from 1996 [3]. Several outbreaks associated with these strains have been documented in USA, South America, Europe, China [4] and in Canada [5]. Furthermore, these strains are endemic in several countries such as Israel, Colombia, Greece, Puerto Rico, and in the East Coast states of the United States [4]. Numerous outbreaks caused by strains other than KPC such as OXA-48 have been reported in Europe [6] and the Middle East [7,8]. The NDM carbapenemase was described in 2009 in Sweden [9] and subsequently identified in India [10], Pakistan [10], England [11], United States [12] and Canada [13–16].

In light of the worldwide emergence of CPE, the Laboratoire de santé publique du Québec (LSPQ) launched a provincial laboratory surveillance program in August 2010. In this study, we present the first molecular and epidemiological report of carbapenem non-susceptible Enterobacteriaceae (CNSE) recovered in the province of Quebec, from 2010 to 2012.

## Materials and Methods

### Study design

From August 2010 to December 2012 (29 months), all clinical laboratories in the province of Quebec were asked to send their carbapenem non-susceptible Enterobacteriaceae (CNSE) isolates to the provincial reference laboratory of Quebec (LSPQ). Analysis of isolates in mandatory surveillance context is part of the mandate of the LSPQ. The isolates selection criteria followed the Clinical and Laboratory Standards Institute (CLSI) carbapenem breakpoints of 2011 [17]. The isolates had to meet at least one of the listed criteria: ertapenem MIC ≥0.5 mg/L (≤22 mm by disk diffusion) and/or imipenem MIC ≥2 mg/L (≤22 mm by disk diffusion) and/or meropenem MIC ≥2 mg/L (≤22 mm by disk diffusion). As *Proteus* spp., *Providencia* spp. and *Morganella* spp. may have elevated imipenem MICs by mechanisms other than carbapenemase production [17], inclusion criteria for these species were based only on ertapenem and meropenem susceptibility results. In July 2012, the inclusion criteria were modified to comply with the most recent CLSI recommendation [18] and inclusion breakpoints became ertapenem MIC ≥1 mg/L (≤21 mm by disk diffusion) and meropenem MIC ≥2 mg/L (≤22 mm by disk diffusion). For *Klebsiella* spp., the inclusion criterion was based on ertapenem MIC only (≥1 mg/L or ≤21 mm by disk diffusion).

### Antimicrobial susceptibility testing

Antibiotic susceptibility was determined by the broth microdilution method following CLSI guidelines [18] for all antibiotics except aztreonam, cefoxitin and tigecycline. For these three antibiotics, MICs were determined using epsilometer test (Etest) following the manufacturer's recommendations (BioMérieux, St-Laurent, QC, Canada). The antibiotics tested were as follows: amikacin, aztreonam, cefepime, cefotaxime, cefoxitin, ceftazidime, ciprofloxacin, colistin, ertapenem, gentamicin, imipenem, meropenem, piperacillin, piperacillin-tazobactam, tigecycline and tobramycin. When available, the MIC results were interpreted according to CLSI criteria. For colistin, the European Committee for Antimicrobial Susceptibility Testing (EUCAST) criteria (http://www.eucast.org/clinical_breakpoints, January 2011) were used. For tigecycline, the criteria supplied by the manufacturer (S ≤2 mg/L, I = 4 mg/L, R ≥8 mg/L) were used (Etest monography, version 2009–09, BioMérieux).

### Phenotypic detection of resistance mechanisms

The modified Hodge test (MHT) was carried out with both ertapenem and meropenem disks according to CLSI guidelines [18]. AmpC and cefoxitin Etests (BioMérieux) were used to detect AmpC production. Metallo-β-lactamase (MBL) production was detected by imipenem MBL Etest (BioMérieux). Detection of extended-spectrum β-lactamase (ESBL) was done using cefotaxime and ceftazidime discs with or without clavulanic acid (MAST Group Ltd., Merseyside, UK) according to CLSI guidelines [18].

### Detection of resistance genes

All isolates non-susceptible to cefoxitin and positive or indeterminate (>32/>32 mg/L) to AmpC Etest were tested by PCR for a plasmid-mediated AmpC gene according to current literature [19]. Three predominant enzyme families were tested: CIT, DHA and FOX. CIT primers enabled amplification of β-lactamases LAT-1, CMY-2 to CMY-7 genes, while DHA primers amplified β-lactamase DHA-1 and DHA-2 genes and FOX primers amplified β-lactamase FOX-1 to FOX-5b genes [20,21]. When detection of carbapenemase genes was negative, bacteria having a chromosomal AmpC, for example *E*. *cloacae*, were considered resistant to carbapenems probably due to AmpC overexpression and porin mutations.

All MHT positive or indeterminate isolates according to CLSI guidelines [18] were tested by PCR. The *bla*_KPC_ gene was detected by PCR primers encompassing the entire coding region of the KPC gene [2]. The KPC negative strains that were carbapenem resistant were sent to the National Microbiology Laboratory (NML) at Winnipeg for detection of other carbapenemases genes by PCR (*bla*_NDM_, *bla*_IMP_, *bla*_VIM_, *bla*_GES_, *bla*_NMC_, *bla*_SME_ and *bla*_OXA-48_) [16].

### Molecular typing by pulsed-field gel electrophoresis (PFGE) and BioNumerics analysis

The genetic relatedness of KPC strains was evaluated by PFGE using the enzyme *Xba*I or *Spe*I according to species. The PFGE fingerprinting patterns were analyzed with BioNumerics software (version 6.5 for Windows, Applied Maths, Kortrijk, Belgium). Band position tolerances and optimization values of 1% were used for all analyses. Similarity coefficient was obtained using Dice coefficients. Cluster analysis was done with the unweighted pair group method with arithmetic averages (UPGMA). Strains were considered to have closely related banding patterns based on molecular typing if their PFGE patterns were ≥90% similar, as determined by the BioNumerics analysis. Patients with more than one bacterial strain carrying KPC (different species or same species with a distinct PFGE pattern) were identified as duplicate (A to S). Nomenclature of KPC PFGE patterns was started before the surveillance program; therefore, some letters are missing in dendrograms. PFGE patterns were designated as follows: each letter represents a different strain and a number indicates the number of band differences between the reference strain and the comparator strain. For example, PFGE pattern A1 has a 1 band difference from pattern A; pattern A1-a, similarly, has a 1 band difference, from pattern A, but this band difference is not the same as that of pattern A1.

## Results

### Patient demographics data of isolates

Samples received at LSPQ for surveillance purposes were accompanied with minimal patient’s information; age, gender, region of residence, sampling date and isolation site. Three hundred and ninety one isolates (52.7%) were collected from males. The mean age was 67 years old (range: <1–100 years) and the median 70 years old. The majority of isolates were from patients aged 60 to 89. Eighty-six percent (n = 638) of the isolates were from the following specimens: urine (n = 235, 31.7%), rectal swabs (n = 189, 25.5%), pus (n = 82, 11.1%), respiratory secretions (n = 75, 10.1%), blood (n = 33, 4.4%) and other sterile fluids (n = 24, 3.2%). Demographic data was unavailable for 50 isolates (6.7%). Information about travel history is not available.

The patient demographics data of KPC strains and specimen types are listed in Table 1. There was no important difference during the 29 months surveillance period for the patient demographics. KPC strains were also mostly (77.5%) retrieved from patients aged ≥ 60 years old. The majority (63.6%) of KPC were from screening specimens (rectal swabs) obtained during the investigation of outbreaks but 10.6% were recovered from urine samples and 21.9% from diverse clinical samples.

**Table 1:** Patients demographics data of KPC strains (n = 151).

Gender	% of strains
Male	57.0
Female	43.0
Age (years)	
0–39	2.0
40–49	9.3
50–59	11.3
60–79	49.0
80–89	27.2
≥ 90	1.3
Specimens types	
Rectal	63.6
Urine	10.6
Pus	6.0
Respiratory	8.6
Blood	4.0
Others	3.3
Unknown	4.0

### Prevalence of carbapenem non-susceptible Enterobacteriaceae

Between August 2010 and December 2012, 1084 isolates were received as part of the surveillance program. A total of 342 isolates (23.4%) were excluded for further analysis as they were duplicate isolates (n = 88) or deemed ineligible (n = 254) i.e. did not meet inclusion criteria. Thus, a total of 742 CNSE isolates were further analysed. Of these 742 isolates, 698 (94%) belonged to 6 species: *Enterobacter cloacae* (356 isolates, 48.0%), *Klebsiella pneumoniae* (142 isolates, 19.1%), *Escherichia coli* (85 isolates, 11.5%), *Serratia marcescens* (46 isolates, 6.2%), *Enterobacter aerogenes* (38 isolates, 5.1%) and *Citrobacter freundii* (31 isolates, 4.2%). The remaining 44 isolates (6%) belonged to 14 different species. The 742 isolates were collected from 672 patients; in 49 patients 2 or more different strains with different PFGE patterns were identified. Change of surveillance selection criteria in July 2012 resulted in a decrease in the number of isolates tested, possibly due to elimination of *E*. *cloacae* isolates with a weak resistance to ertapenem (MIC of 0.5 mg/L).

### Carbapenem resistance mechanisms

The distribution of the different carbapenemases observed among the Enterobacteriaceae isolates is summarized in Table 2. Carbapenemases were responsible for resistance in one hundred sixty nine isolates (169, 22.8%). Of these, 151 (89%) were KPC and 18 (11%) produced carbapenemases other than KPC (SME (n = 9), OXA-48 (n = 5), NDM (n = 3), and NMC (n = 1)). The distribution of carbapenemase genes among Enterobacteriaceae species is shown in Table 3. The most prevalent species among the KPC strains were *K*. *pneumoniae* (49.0%) and *E*. *cloacae* (19.9%).

**Table 2:** Distribution of carbapenem non-susceptible Enterobacteriaceae (CNSE) over the surveillance program.

	2010^a^	2011	2012^b^	TOTAL
Total of isolates	127	390	225	742
No. of CPE isolates (%)	38 (29.9)	79 (20.3)	52 (23.1)	169 (22.8)
KPC (%)	36 (28.3)	74 (19.0)	41 (18.2)	151 (20.4)
SME (%)	1 (0.8)	2 (0.5)	6 (2.7)	9 (1.2)
OXA-48 (%)	0	2 (0.5)	3 (1.3)	5 (0.7)
NDM (%)	1 (0.8)	0	2 (0.9)	3 (0.4)
NMC (%)	0	1 (0.3)	0	1 (0.1)
No. of CNSE non-CPE (%)	89 (70.1)	311 (79.7)	173 (76.9)	573 (77.2)

^a^ Surveillance program was started on August 2010.

^b^ Modification of inclusion criteria on July 2012.

CPE: carbapenemase-producing Enterobacteriaceae.

**Table 3:** Distribution of carbapenemase genes among Enterobacteriaceae species.

Species	No. of strains per year			Total
	2010	2011	2012	
KPC	36	74	41	151
*K*. *pneumoniae*	26	35	13	74
*E*. *cloacae*	3	17	10	30
*S*. *marcescens*	3	7	3	13
*E*. *coli*	2	7	4	13
*K*. *oxytoca*	1	4	3	8
*C*. *freundii*	1	3	3	7
*C*. *koseri*	0	0	3	3
*C*. *youngae*	0	1	1	2
*K*. *ascorbata*	0	0	1	1
NDM	1	0	2	3
*K*. *pneumoniae*				
OXA-48	0	2	3	5
*K*. *pneumoniae*	0	2	1	3
*E*. *coli*	0	0	2	2
SME	1	2	6	9
*S*. *marcescens*				
NMC	0	1	0	1
*E*. *cloacae*				
TOTAL	38	79	52	169

A total of 573 strains (77.2%) were presumed resistant to carbapenems by mechanisms other than carbapenemase production. More than half of these strains (57%) were *E*. *cloacae*. Overall, the most frequent carbapenem resistance mechanism was most likely AmpC overexpression associated with porin mutations (416 strains, 56.1%). Of these, 323 (77.6%) were *E*. *cloacae*. Other strains (n = 93) with this potential mechanism are species other than *E*. *cloacae* and are known as have an intrinsic AmpC. In 21.1% (n = 157) of the 742 isolates, carbapenem resistance was due to mechanisms other than carbapenemase or AmpC overexpression. The most frequent mechanisms included extended-spectrum β-lactamase production combined with porin mutations (n = 50, 6.7%), plasmid-mediated AmpC production combined to porin mutations (n = 36, 4.9%) or efflux mechanism and/or porin mutations (n = 52, 7.0%). The most prevalent plasmid-mediated AmpC β-lactamase was CIT (26/36, 72%). Detection of AmpC overexpression, porin mutations and efflux mechanism were not confirmed by laboratory analysis.

### Pulsed field gel electrophoresis of KPC strains

**Fig 1:**
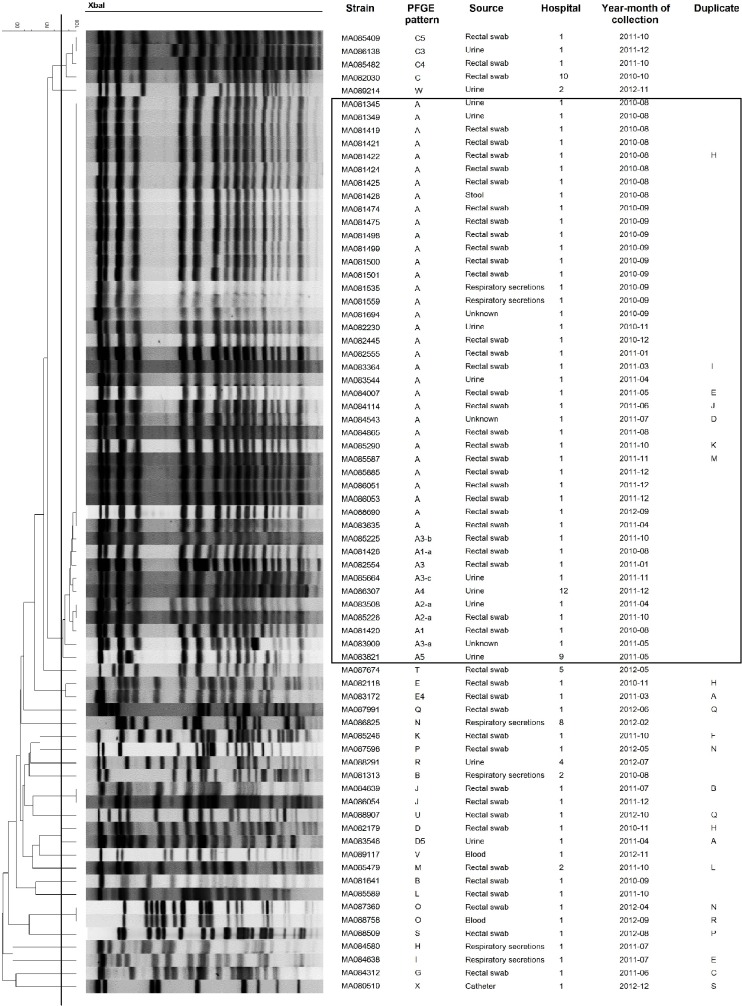
PFGE patterns of *K*. *pneumoniae* KPC strains (n = 73). One strain (MA084240) was untypable by *Xba*I. Black line in dendrogram represents percentage similarity cut-off. Box represents the main cluster within the species. Patients with more than one bacterial strain carrying KPC gene were identified as duplicate, following the same nomenclature through all figures.

**Fig 2:**
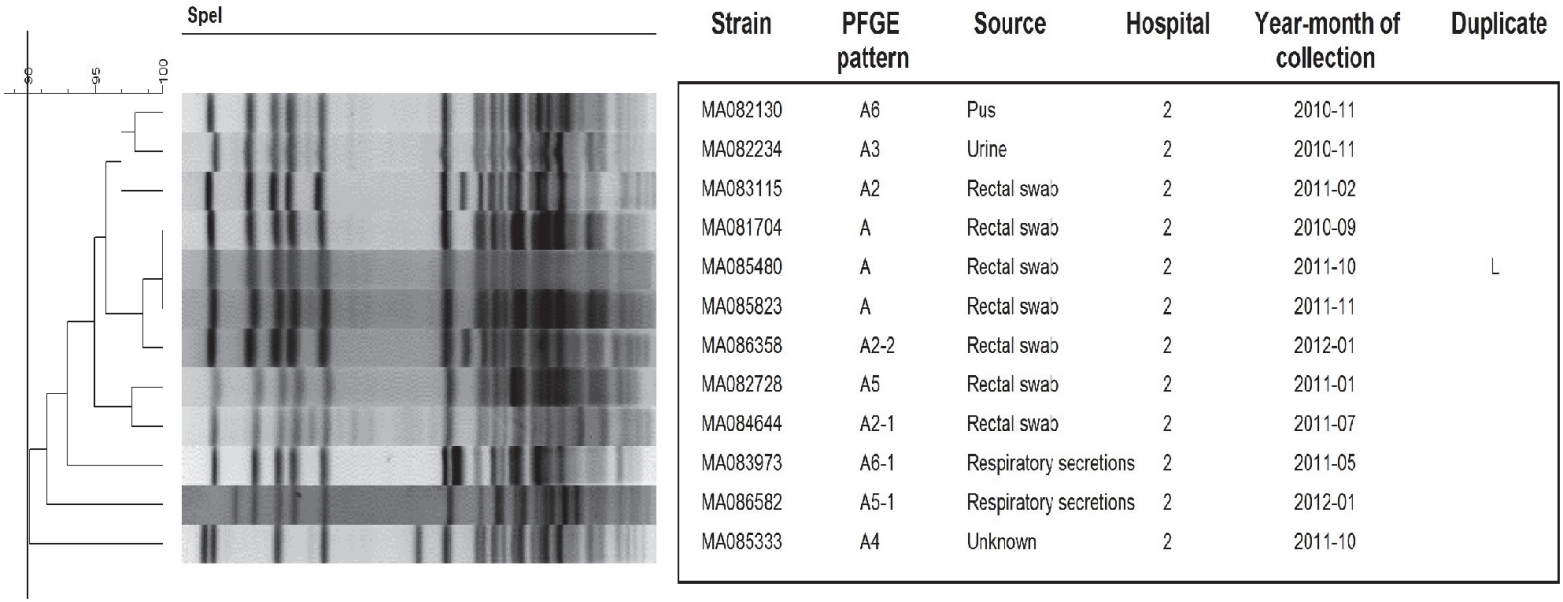
PFGE patterns of *S*. *marcescens* KPC strains (n = 12). One strain (MA088423) was untypable by *Spe*I. Black line in dendrogram represents percentage similarity cut-off. Box represents the main cluster within the species. Patients with more than one bacterial strain carrying KPC gene were identified as duplicate, following the same nomenclature through all figures.

**Fig 3:**
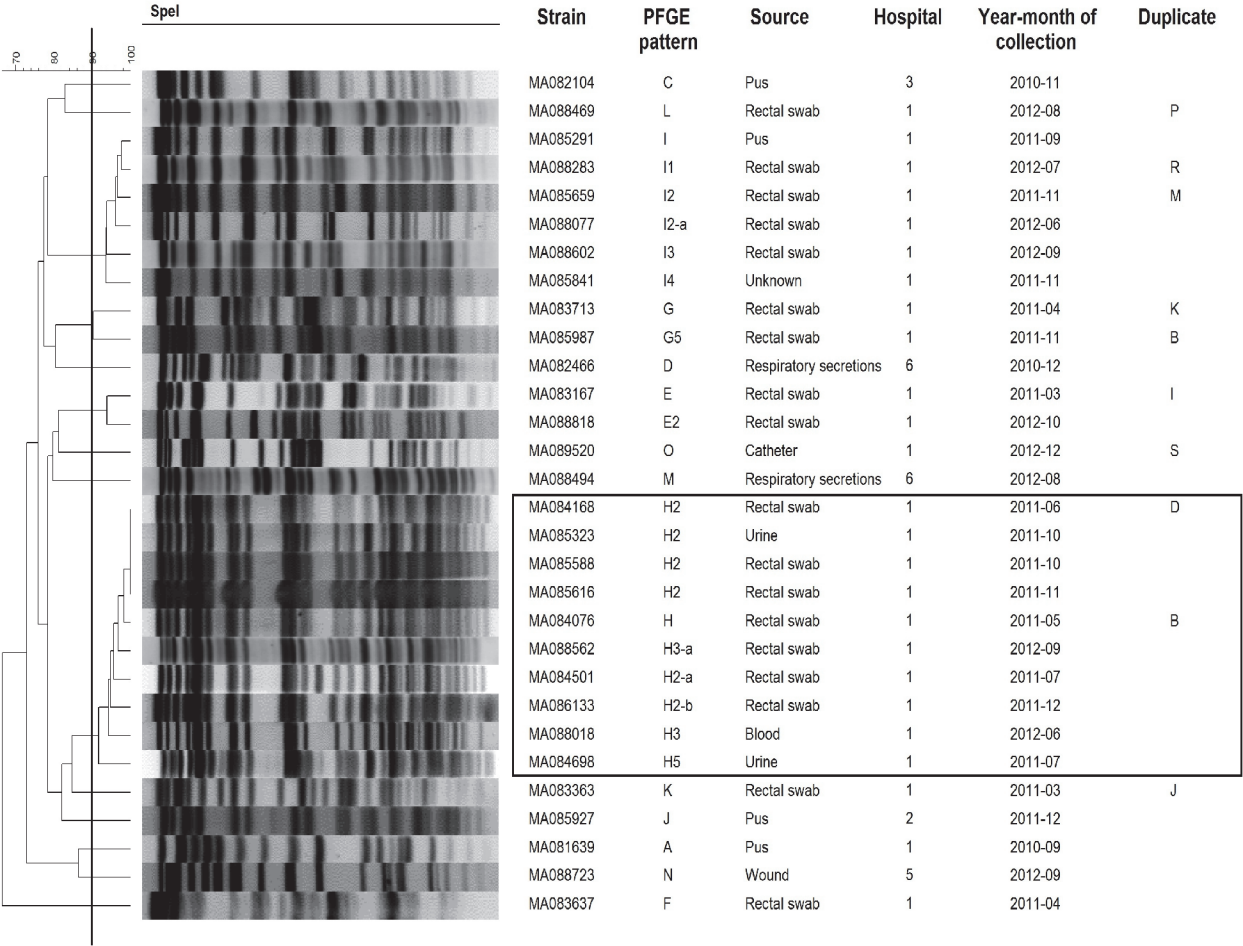
PFGE patterns of *E*. *cloacae* KPC strains (n = 30). Black line in dendrogram represents percentage similarity cut-off. Box represents the main cluster within the species. Patients with more than one bacterial strain carrying KPC gene were identified as duplicate, following the same nomenclature through all figures.

Using PFGE, 73 different patterns and related pattern were obtained from 151 KPC strains. The number of unique patterns varied among different species: *K*. *pneumoniae* (n = 24), *E*. *cloacae* (n = 14), *E*. *coli* (n = 12), *K*. *oxytoca* (n = 8), *C*. *freundii* (n = 7), *C*. *koseri* (n = 3), *S*. *marcescens* (n = 2), *C*. *youngae* (n = 2) and *K*. *ascorbata* (n = 1). Although, KPC strains were isolated in 12 different hospitals the majority were from 2 hospitals (88%) (n = 110 and n = 23, respectively). Three major clusters were associated with nosocomial outbreaks in 2 Montreal hospitals: *K*. *pneumoniae* (n = 41), *S*. *marcescens* (n = 12) and *E*. *cloacae* (n = 10) (Figs 1–3). These outbreaks represented approximately 42% of KPC strains. The remaining species comprise strains that were not clonally related, all exhibiting <90% similar PFGE patterns with the exception of two *E*. *coli* strains (S1–S4 Figs). Multiple unrelated organisms harbouring KPC (n = 49) were isolated from 19 patients over the study period. Eleven patients harboured 2 strains, 6 patients harboured 3 strains, 2 patients harboured, respectively, 4 and 5 strains. In 16/19 of these patients bacteria from multiple species were isolated. For 5 patients (26.3%), two strains or more were isolated from the sites sampled on the same date.

### Modified Hodge test

Among all isolates tested, the MHT was positive for all KPC strains tested (n = 140) with both ertapenem (ERT) and meropenem (MEM) disks. The 3 *K*. *pneumoniae* strains harbouring NDM were positive with ERT disks and negative with MEM disks. For strains of *S*. *marcescens* carrying the *bla*_SME_ gene, eight isolates were positive and one negative with ERT disks. Conversely, six isolates were positive and three negative using MEM disks. All OXA-48 and NMC strains were positive using both ERT and MEM disks. Strains resistant to carbapenems by mechanisms other than carbapenemase production gave variable results: 49.2% and 26.4% were positive and 50.8% and 73.6% were negative for MHT using ERT and MEM disks respectively.

MHT with ERT disks was a highly sensitive (99.4%) but non-specific indicator (50.8%) for CPE detection. MHT with MEM disks was a better indicator for CPE detection (sensitivity 96.2% and specificity 73.5%). The positive predictive value (PPV) and negative predictive value (NPV) for carbapenemase detection were 36.1% and 99.7% for MHT with ERT disks and 50.5% and 98.6% for MHT with MEM disks, respectively.

### Antimicrobial susceptibilities of CPE

Although antimicrobial susceptibilities were performed for all KPC strains, data for 93 strains are shown (Table 4). Only KPC strains with unique PFGE pattern and/or distinct antibiotic susceptibility profiles were shown to avoid data skew due to numerous strains related to outbreaks. Ninety-eight percent of KPC strains were susceptible to tigecycline while 99% were resistant to ertapenem, 95% to imipenem, 87% to meropenem, and 97% to aztreonam. Approximately 30% of the strains were resistant to colistin and 44% exhibited resistance to ciprofloxacin. Indeed, 56% of isolates was resistant to an aminoglycoside. All isolates were resistant to at least one cephalosporin (Table 4).

**Table 4:** Antimicrobial susceptibilities of strains carrying *bla*_KPC_, *bla*_NDM_, *bla*_OXA-48_ and *bla*_SME_ genes.

Antibiotics	*bla*_KPC_ (n = 93)^a^				*bla*_NDM_ (n = 3)				*bla*_OXA-48_ (n = 5)				*bla*_SME_ (n = 9)			
	Range	MIC_50_	MIC_90_	%R	Range	MIC_50_	MIC_90_	%R	Range	MIC_50_	MIC_90_	%R	Range	MIC_50_	MIC_90_	%R
Ertapenem	1 - >32	32	>32	99	>32 - >32	>32	>32	100	1 - >32	4	>32	80	2 - >32	>32	>32	100
Imipenem	1 - >32	16	>32	95	16 - >32	16	>32	100	1 - >32	4	>32	80	>32 - >32	>32	>32	100
Meropenem	0.25 - >32	16	32	87	>32 - >32	>32	>32	100	0.25 - >32	2	>32	20	4 - >32	>32	>32	100
Cefotaxime	8 - >32	>32	>32	100	>32 - >32	>32	>32	100	0.5 - >32	4	>32	60	0.25–1	0.5	1	0
Ceftazidime	4 - >64	>64	>64	99	>64 - >64	>64	>64	100	0.12 - >64	1	>64	20	0.25–1	0.25	1	0
Cefepime	2 - >64	>64	>64	85	64 - >64	>64	>64	100	0.25 - >64	0.5	>64	20	≤0.06–0.25	0.12	0.25	0
Cefoxitin^b^	2 - ≥256	32	≥256	63	≥256 - ≥256	≥256	≥256	100	4–64	8	64	20	8 - ≥256	32	64	78
Amikacin	0.25 - >64	4	32	6	>128 - >128	>128	>128	100	2–16	2	16	0	0.5–8	4	4	0
Gentamicin	0.25 - >64	8	32	43	>64 - >64	>64	>64	100	0.5 - >64	1	>64	40	0.5–2	1	2	0
Tobramycin	0.5 - >64	4	64	40	>64 - >64	>64	>64	100	1 - >64	16	>64	60	0.5–8	2	4	0
Ciprofloxacin	≤0.06 - >64	2	>64	44	64 - >64	>64	>64	100	≤0.06 - >64	0.5	>64	40	≤0.06–0.5	0.12	0.5	0
Aztreonam^b^	2 - ≥256	≥256	≥256	97	0.12–64	64	64	67	0.06 - ≥256	0.12	≥256	20	2–16	4	16	44
Colistin	1 - >64	2	4	31^c^	2–4	2	4	33	2–4	4	4	60	>64 - >64	>64	>64	100
Tigecycline^b^	0.12–16	1	4	2	1–4	2	4	0	0.12–2	0.5	2	0	1–4	2	4	0

%R: percentage of resistance.

Range, MIC_50_ and MIC_90_: minimum inhibitory concentration in mg/L.

^a^ Data presented for strains with unique PFGE pattern and/or unique antibiotics susceptibility profile.

^b^ Data presented for 92 strains; no growth for one *S*. *marcescens* strain on Mueller-Hinton agar for Etest testing.

^c^ *S*. *marcescens* is intrinsically resistant to colistin. This data was therefore not included in calculating the overall % colistin resistance in KPC strains.

Antibiotics susceptibility testing for NDM, OXA-48 and SME are presented in Table 4. All NDM strains were resistant to all antibiotics tested except to tigecycline (0%), colistin (33%) and aztreonam (67%). OXA-48 strains were generally less resistant to antimicrobials than KPC and NDM strains. All SME and NMC strains were resistant to all carbapenems but sensitive to cephalosporins, aminoglycosides, ciprofloxacin and tigecycline.

## Discussion

The emergence of NDM-1 strains in India, Pakistan and England [10] prompted the LSPQ to establish a province-wide laboratory-based surveillance program to study and follow the emergence of CPE in Quebec. From 2010 to 2012, carbapenem resistance in Enterobacteriaceae isolates from Quebec has been relatively stable. However, some variations were noted but this was mainly due to a reduction of *K*. *pneumoniae* PFGE pattern A strains harbouring KPC (58% in 2010, 28% in 2011 and 2% in 2012). Overall, we noted a decrease of CPE isolates from 30% to 23%. This was most likely due, for the most part, to a decrease or the resolution of CPE outbreaks which reduced their size and length over the study period. As well, the inclusion criteria of surveillance program were changed in 2012.

The CLSI [18] changed the carbapenem MIC breakpoints to increase the specificity of carbapenemase detection. In July 2012, inclusion criteria of the surveillance program were modified according to CLSI in order to concentrate our efforts on relevant isolates. The new algorithm aimed to target CPE rather than carbapenem resistant isolates due to other mechanisms, primarily by overexpression of chromosomal AmpC possibly combined with a decrease in membrane permeability, a mechanism commonly found in *E*. *cloacae* strains. More specifically, the goal was to eliminate *E*. *cloacae* strains with an ertapenem MIC of 0.5 mg/L that are normally non-CPE. From the beginning of the surveillance, only one KPC strain, an *E*. *cloacae* with low carbapenems MICs was detected (ertapenem 1 mg/L, imipenem 1 mg/L and meropenem 0.25 mg/L). The modification of inclusion criteria suggests that few KPC stains with low carbapenems MICs might have been missed. In our study, MICs for those antibiotics were generally high for KPC strains but MIC may vary [4,22].

Carbapenemases are the focus of worldwide attention due to the rapid dissemination [4]. USA, Europe, India and China are known as endemic countries. Three main carbapenemases are reported all around the world: KPC, NDM and OXA-48. KPC strains are mostly found in the USA, Israel, Greece and Italy. The Indian subcontinent is recognized as a NDM endemic zone. OXA-48 is often recovered in Mediterranean area and North Africa. Verona integron–encoded metallo-β-lactamase (VIM) also has been reported worldwide with a higher prevalence in southern Europe (Greece) [4]. In this survey, 22.8% of isolates were CPEs while 77.2% of isolates were resistant to carbapenems by a mechanism other than the production of carbapenemases. The most frequent type of CPE detected was KPC (89.3%) although a small number of isolates SME (5.3%) and OXA-48 (3.0%) were also detected. During the study period, only three NDM strains were detected. Even if CPE were found in Canada, our country is not recognized as endemic. KPC are the most common carbapenemases identified in Canada. Other Canadian provinces have identified carbapenemase-producing strains. KPC strains have been isolated in all provinces except New Brunswick, Nova Scotia, Prince Edward Island, and Saskatchewan; however, the majority were found in the province of Quebec (n = 153; 72.9%) (Michael Mulvey, personal communication). In contrast, NDM strains were only found in British Columbia, Alberta, Ontario, Quebec, and New Brunswick with the largest proportion recovered in Ontario (n = 55; 23.8%) and British Columbia (n = 25; 28.7%) (Michael Mulvey, personal communication) where nosocomial [23,24] and community outbreaks have been described. Few KPC, NDM and OXA-48 strains were found in the rest of the Canada (Michael Mulvey, personal communication and Mataseje *et al*. [25]. In our study, KPC were found in 9 different species including in *Kluyvera ascorbata* and, to our knowledge, this is the first report of KPC in this species in Canada.

The key to prevent and control outbreaks of carbapenemase-producing strains in healthcare-associated settings is to have reliable and rapid phenotypic tests or confirmatory methods such as PCR to confirm carbapenemase-producing strains. Moreover, aggressive infection-control measures are essential to minimize spread of these bacteria. Thus, surveillance programs are essential to detect CPE and prevent their dissemination. In addition to epidemiological investigation, PFGE analyses indicate occurrence of an outbreak. However, distinct PFGE patterns do not necessarily indicate that a situation is under control because KPC plasmids or KPC transposons can be spread horizontally. Moreover, multiple Enterobacteriaceae species harbouring KPC complicates outbreak investigations. We found an important number of patients (15.7%) with 2 to 5 distinct KPC strains. This finding not only complicates the provincial portrait of CPE but makes outbreak investigations difficult. Therefore, diversity of KPC strains found in the same patient shows the possibility of KPC gene transmission in the same species or in different ones via KPC plasmid or KPC transposon. A recent study revealed that *E*. *cloacae* strains with nonclonal genetic background share the same KPC plasmid and highlights the complexity of a KPC outbreak [5]. Such multispecies outbreaks of Gram-negative bacteria require methods which allow a deeper investigation of the molecular mode of dissemination such as whole genome sequencing.

For clinical laboratories, detection of some carbapenemases such as SME, OXA-48 and NMC represents a major challenge due to difficulties with the interpretation of results obtained by conventional phenotypic methods. Those enzymes hydrolyse carbapenems at lower level than KPC and are not strongly positive to modified Hodge test. The MHT have a high sensitivity for KPC detection but less for other carbapenemase genes. However, specificity is low due to overexpressed AmpC strains which lead to false positive results. In addition, SME and NMC showed very weak activity against expanded-spectrum cephalosporins. Only molecular techniques can definitively confirm the presence of genes encoding for these carbapenemases. However, contrary to SME which is recognized as a chromosomal carbapenemase, dissemination of OXA-48 could be an issue due to plasmid localization [26]. Indeed, OXA-48 outbreaks were recently reported in Spain, Greece, Tunisia and France [27–30] and European dissemination of a single OXA-48-producing *Klebsiella pneumoniae* clone was described [8].

In summary, this study highlights the presence of KPC in the province of Quebec. During the study period, three KPC outbreaks were described in two hospitals in the Montreal area. Worldwide, *K*. *pneumoniae* KPC strains were often identified as ST-258 clone [31–33] indicated that the potential for carbapenemases to persist and spread globally. Thus, continuous surveillance of CNSE should be maintained considering also recent outbreaks caused by NDM [23,34,35] and those caused by KPC in Quebec [5,36]. Due to rapid KPC, OXA-48 and NDM dissemination and outbreaks reported elsewhere, it is crucial to maintain active surveillance of CPE. Tracking of OXA-48 and NDM is also very important due to their importance in Europe. Molecular methods are essential for the specific determination of resistance profile and for outbreak investigation. Since therapeutic options are limited to treat infections due to carbapenemase-producing strains and generally have a poor clinical outcome, monitoring antibiotic resistance of these strains is crucial.

Analysis of 742 carbapenem non-susceptible isolates highlighted several concerns. (i) The two main organisms with decreased susceptibility to carbapenems are *K*. *pneumoniae* and *E*. *cloacae*. However, a major difference was observed between these two groups: 56% of *K*. *pneumoniae* isolates produced a carbapenemase, compared to only 9% of *E*. *cloacae*. (ii) The main carbapenemase identified was KPC, which could be partially linked to dissemination in the hospital. Only two antibiotics remained effective against most but not all KPC strains: 69% were susceptible to colistin and 86% to tigecycline. The PCR has been shown to be a powerful technique to discriminate rapidly between carbapenemase and non-carbapenemase producers.

## Supporting Information

S1 Fig PFGE patterns of *K. oxytoca* KPC strains (n = 8). Black line in dendrogram represents percentage similarity cut-off. Patients with more than one bacterial strain carrying KPC gene were identified as duplicate, following the same nomenclature through all figures.
(TIF)

S2 Fig PFGE patterns of *C. freundii* KPC strains (n = 7). Black line in dendrogram represents percentage similarity cut-off. Patients with more than one bacterial strain carrying KPC gene were identified as duplicate, following the same nomenclature through all figures.
(TIF)

S3 Fig PFGE patterns of *C. koseri* KPC strains (n = 3). Black line in dendrogram represents percentage similarity cut-off. Patients with more than one bacterial strain carrying KPC gene were identified as duplicate, following the same nomenclature through all figures.
(TIF)

S4 Fig PFGE patterns of *E. coli* KPC strains (n = 13). Black line in dendrogram represents percentage similarity cut-off. Patients with more than one bacterial strain carrying KPC gene were identified as duplicate, following the same nomenclature through all figures.
(TIF)
