# Supplementary figures and images for: Carbapenem Non-Susceptible Enterobacteriaceae in Quebec, Canada: Results of a Laboratory Surveillance Program (2010–2012)
Source: PLoS One. 2015 Apr 24;10(4):e0125076. doi: 10.1371/journal.pone.0125076 (PMC4409364; doi:10.1371/journal.pone.0125076)

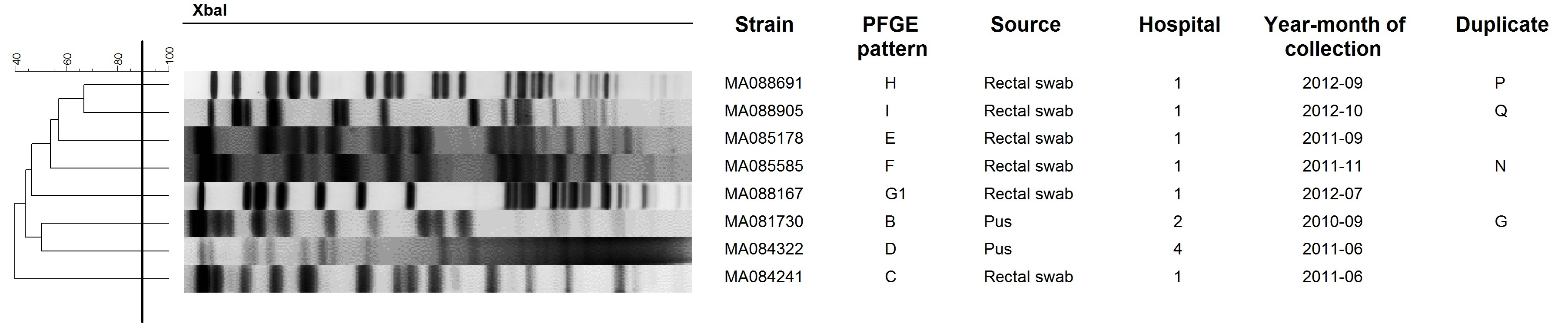

Supplement: S1 Fig — Black line in dendrogram represents percentage similarity cut-off. Patients with more than one bacterial strain carrying KPC gene were identified as duplicate, following the same nomenclature through all figures. (TIF) [file pone.0125076.s001.tif]

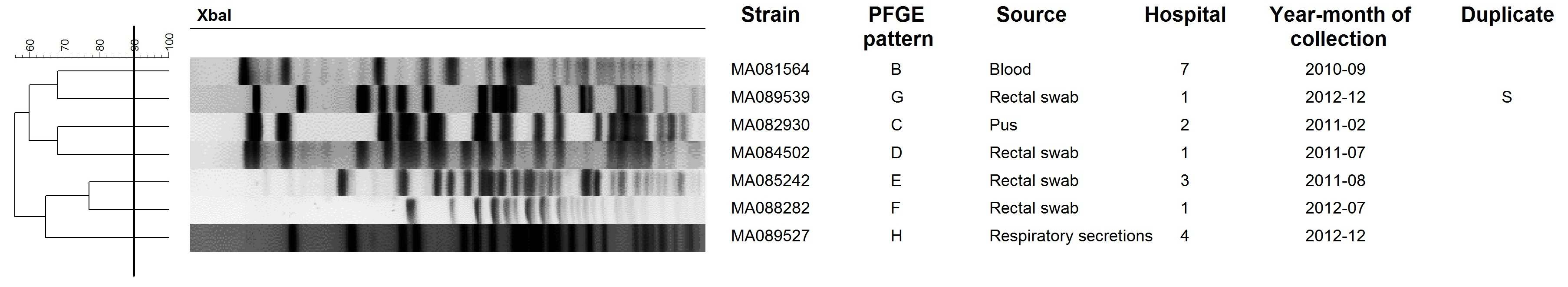

Supplement: S2 Fig — Black line in dendrogram represents percentage similarity cut-off. Patients with more than one bacterial strain carrying KPC gene were identified as duplicate, following the same nomenclature through all figures. (TIF) [file pone.0125076.s002.tif]

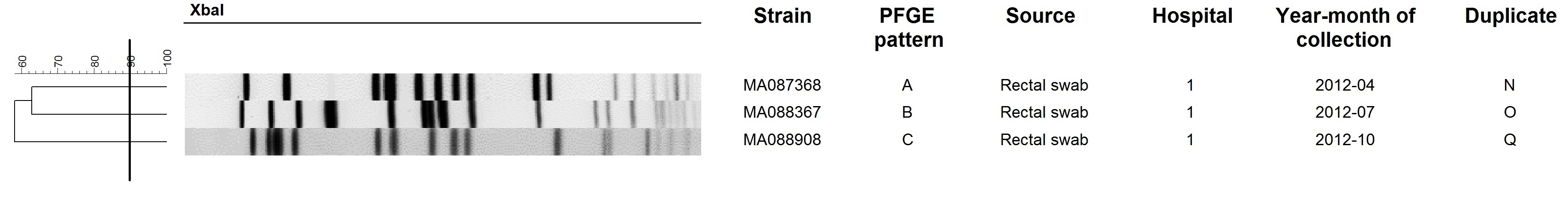

Supplement: S3 Fig — Black line in dendrogram represents percentage similarity cut-off. Patients with more than one bacterial strain carrying KPC gene were identified as duplicate, following the same nomenclature through all figures. (TIF) [file pone.0125076.s003.tif]

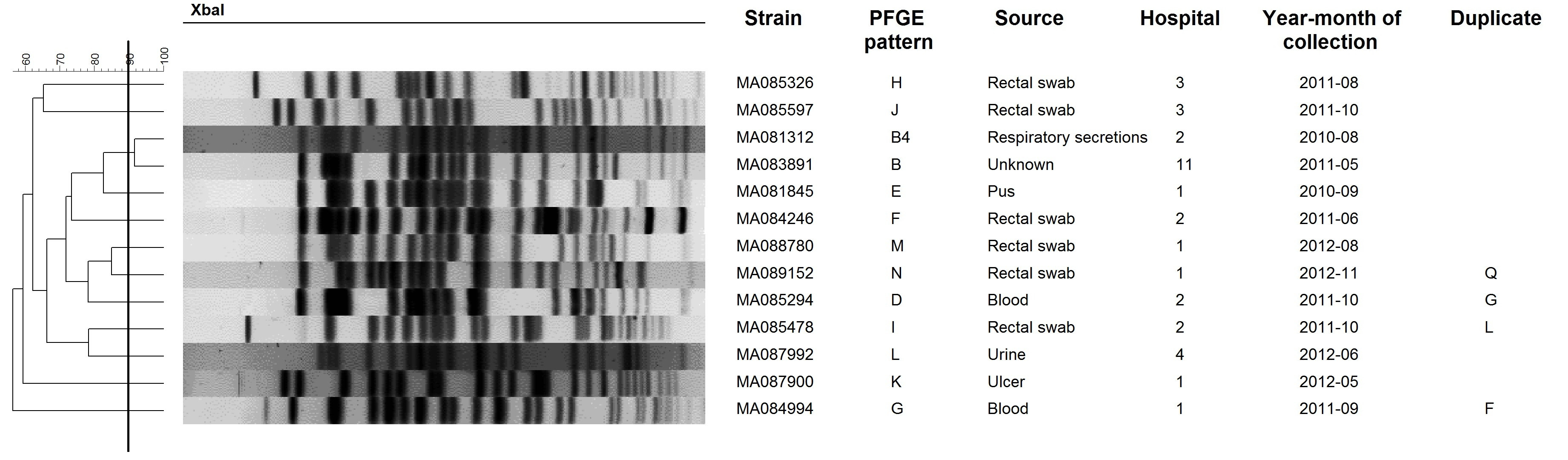

Supplement: S4 Fig — Black line in dendrogram represents percentage similarity cut-off. Patients with more than one bacterial strain carrying KPC gene were identified as duplicate, following the same nomenclature through all figures. (TIF) [file pone.0125076.s004.tif]
